# Effect of Probiotic (VSL#3) and Omega-3 on Lipid Profile, Insulin Sensitivity, Inflammatory Markers, and Gut Colonization in Overweight Adults: A Randomized, Controlled Trial

**DOI:** 10.1155/2014/348959

**Published:** 2014-03-26

**Authors:** Hemalatha Rajkumar, Naseha Mahmood, Manoj Kumar, Sudarshan Reddy Varikuti, Hanumanth Reddy Challa, Shiva Prakash Myakala

**Affiliations:** Department of Microbiology and Immunology, National Institute of Nutrition, ICMR, Hyderabad 500007, India

## Abstract

To evaluate the effects of probiotic (VSL#3) and omega-3 fatty acid on insulin sensitivity, blood lipids, and inflammation, we conducted a clinical trial in 60 overweight (BMI > 25), healthy adults, aged 40–60 years. After initial screening the subjects were randomized into four groups with 15 per group. The four groups received, respectively, placebo, omega-3 fatty acid, probiotic VSL#3, or both omega-3 and probiotic, for 6 weeks. Blood and fecal samples were collected at baseline and after 6 weeks. The probiotic (VSL#3) supplemented group had significant reduction in total cholesterol, triglyceride, LDL, and VLDL and had increased HDL (*P* < 0.05) value. VSL#3 improved insulin sensitivity (*P* < 0.01), decreased hsCRP, and favorably affected the composition of gut microbiota. Omega-3 had significant effect on insulin sensitivity and hsCRP but had no effect on gut microbiota. Addition of omega-3 fatty acid with VSL#3 had more pronounced effect on HDL, insulin sensitivity and hsCRP. Subjects with low HDL, insulin resistance, and high hsCRP had significantly lower total lactobacilli and bifidobacteria count and higher *E. coli* and bacteroides count.

## 1. Introduction

Inflammatory mediators have been recognized as factors that increase the risk of insulin resistance, diabetes, and cardiovascular diseases. Inflammation is also associated with metabolic syndrome, which in turn increases the risk of coronary heart disease [1–3]. Forty-four percent of the diabetes burden, 23% of the ischaemic heart disease burden, and between 7% and 41% of certain cancer burdens are attributable to overweight and obesity [4]. In India, 12.6% of women and 9.3% of men are obese [5]. 

Lipids and lipoproteins are well known risk factors for ischemic heart disease. Elevated levels of triglyceride, cholesterol, and LDL are documented as risk factors for atherogenesis. It is noteworthy that CRP plasma levels even slightly higher from the conventional upper limit of normal (1 mg/dL) have been associated with a 2-3-fold increase in risk of future myocardial infarction, stroke, and peripheral atherosclerosis among apparently healthy middle-aged men and women [6]. The association between CRP and cardiovascular disease is more than a mere epiphenomenon; in other words, this acute-phase reactant is not just a marker of increased inflammatory activity, but it is also directly involved in the pathogenesis of atherothrombosis through several mechanisms and is associated with several cardiovascular risk factors, such as age, smoking, hypertension, exercise, plasma lipids, homocysteine, and body mass index (BMI) [7, 8]. CRP has been suggested to be higher both in overweight (BMI 25–29.9 kg/m^2^) and obese (BMI ≥ 30 kg/m^2^) patients than in normal weight (BMI < 25 kg/m^2^) subjects and has been attributed to be mediated by elevated cytokines, such as interleukin-6 (IL-6) and tumor necrosis factor a (TNF-*α*), which are both expressed in adipose tissue [9–13].

Past studies have demonstrated that probiotics could improve lipid disorders by lowering blood cholesterol levels and increasing resistance of low density lipoprotein to oxidation [14, 15]. A combination of probiotic strains was also found to reduce the onset of insulin resistance and diabetes in animals [16]. VSL#3 is a commercially available mixture of probiotics containing a high concentration (450 billion colonies/sachet) of viable, lyophilized bifidobacteria,lactobacilli, and* Streptococcus thermophilus*. Li et al. [16] found that dietary supplementation with VSL#3 improved hepatic insulin resistance in diabetic ob/ob mice after 4 weeks of treatment. These findings provided the rationale for our present study, in which we tested the effectiveness of VSL#3 on inflammation, lipid profile, and insulin sensitivity in apparently healthy overweight human subjects. Omega-3 has also been found to improve obesity-induced metabolic syndrome through regulating chronic inflammation [17–19]. Therefore in the present study, we compared the effects of probiotics with omega-3 fatty acid, which is well known for its anti-inflammatory properties and cholesterol lowering effect [20].

Thus, this study investigated whether VSL#3 alone or with omega-3 as adjunct improved lipid profile, insulin sensitivity, and inflammatory responses which are indicators of risk for metabolic syndrome and, ultimately, heart disease, diabetes, and stroke, in a healthy overweight population.

## 2. Materials and Methods

The study protocol was approved by the Institutional Ethics Committee (IEC), Indian Council of Medical Research (ICMR). The trial was registered under Clinical Trials Registry India (CTRI/2012/08/002856) (ICMR). Before entering the study, the subjects gave their written informed consent. All clinical investigations were conducted according to the principles expressed in declaration of Helsinki.

Apparently healthy adult volunteers of both sexes, aged between 40 and 60 years and with BMI > 25, were recruited. Those with hypertension, diabetes mellitus, any metabolic disorder, acute gastrointestinal disorders, pregnancy or lactation, alcohol consumption, smoking, antibiotic therapy, or use of related medications before and during the 6-week study period were excluded.

The study was a randomized, placebo-controlled trial. The volunteers were randomly assigned to receive either placebo (*n* = 15), VSL#3 capsules (*n* = 15), omega-3 fatty acid capsules (*n* = 15), or omega-3 capsule + VSL#3 capsule (*n* = 15), for 6 weeks. They were asked not to consume any other probiotic-containing products (from a supplied list) during the study. Subjects were given an identification number and were assigned a treatment code by a scientist blind to the treatments corresponding with the codes. The probiotic and placebo groups could be blinded but it was not possible to blind subjects or field staff to the omega-3 supplementations, as the capsules looked different. Nevertheless, all the investigators, including the medical doctor collecting clinical data and those collecting anthropometric measurement, the laboratory technician (who carried out all the biochemical parameters and caecal colonization), and the statistician, were blind to the treatment. After completion of the biochemical and statistical analysis, the groups were decoded. Hence, there was minimum chance of bias entering the study results or interpretation.

The placebo and VSL#3 groups were given identical-looking coded wraps containing either placebo or probiotic and were instructed to take one capsule every day before any meal. The third group was advised to take one capsule of omega-3 fatty acid before breakfast, and the fourth group was advised to take one omega-3 capsule and one probiotic capsule before breakfast.

### 2.1. Probiotics (VSL#3), Omega-3, and Placebo

VSL#3 (manufactured in India by Sun Pharmaceutical Ind. Ltd.) is a freeze-dried pharmaceutical probiotic preparation containing 112.5 × 10^9^ CFU/capsule of three strains of bifidobacteria (*Bifidobacterium longum*,* Bifidobacterium infantis*,* and Bifidobacterium breve*), four strains of lactobacilli (*Lactobacillus acidophilus*,* Lactobacillus paracasei*, Lactobacillus* delbrueckii *subsp.* bulgaricus*, and* Lactobacillus plantarum*), and one strain of* Streptococcus salivarius *subsp.* thermophilus*. Identical-looking placebo capsules containing 40 mg microcrystalline cellulose were used for blinding. The capsules were stored at 2 to 8°C prior to distribution and the subjects were instructed to refrigerate the capsules. Bacterial viability was confirmed at the Department of Microbiology and Immunology, National Institute of Nutrition (ICMR), Hyderabad, by plating serial dilutions of bacterial suspensions onto* Bifidobacterium* Agar,* Lactobacillus* MRS Agar (HiMedia, India), and* Streptococcus thermophilus* Agar (HiMedia Laboratories Pvt. Ltd., India) followed by incubation in an anaerobic jar with anaerobic gas pack (HiMedia, India) at 37°C for 48 hours. The omega-3 capsule contained 180 mg EPA and 120 mg DHA per capsule (Dr. Reddy's Laboratories Ltd. Hyderabad, India).

### 2.2. Biochemical Analyses

Blood samples were drawn at baseline and after 6 weeks of intervention (study end), after an overnight fast. Inflammatory markers such as IL-1 *β*, TNF-*α*, and IL-6 assays were carried out using ELISA. Measurements of all the serum parameters were done in duplicate and mean concentrations were calculated.

Serum glucose was estimated using Serum Glucose Biosystems kit (Barcelona, Spain). Total cholesterol, triglycerides, and high-density lipoprotein were measured using kits from Lab Care Diagnostics (India) Pvt. Ltd. The Friedewald equation [21] was applied to analyse all the lipid fractions. HOMA was used to evaluate insulin resistance before and after treatment [22]. Serum high-sensitivity C-reactive protein (hs-CRP) was estimated by dbc-hs Krishgen, Biosystems (India) CRP kit.

### 2.3. Stool Sampling and Colony Counting

Fecal samples were obtained from the subjects at the beginning of the trial and after 45 days, for stool culture. The specimens were collected in sterile plastic containers and were immediately preserved at 4°C and were analysed on the same day or within 2 days. Fecal samples were homogenized using PBS and serially diluted. Plates were incubated in triplicate using selective media for enumeration of total aerobes (Nutrient agar, HiMedia India), total anaerobes (Schaedler agar, HiMedia India), coliforms (Violet Red Bile Agar, HiMedia India),* E. coli* (Eosin Methylene blue Agar, HiMedia India), bacteroides (Bacteroides Bile Esculin Agar Base, HiMedia India), bifidobacteria (*Bifidobacterium* Agar, HiMedia), lactic acid bacteria (*Lactobacillus* MRS Agar, HiMedia India), and* Streptococcus thermophilus* (*Streptococcus thermophilus* Agar, HiMedia India). Plates were incubated aerobically or anaerobically as appropriate and the colonies were counted after 48 hours.

### 2.4. Statistical Analysis

Assuming that the probiotics and omega-3 supplementation would reduce hsCRP concentration, a sample size of 15 in each arm was calculated to detect a 20% reduction in hsCRP with treatment, with a power of 80% and 5% significance using power and Sample Size Calculation software, version 3.0.14. Variation between groups at baseline was evaluated by one-way ANOVA. Changes from baseline to endline after treatment were evaluated by applying a one-way ANOVA. Repeated-measures analysis of variance (ANOVA) was used to determine if there were treatment and/or time differences. When there was a significant difference, one-way ANOVA was used followed by Newman-Keuls multiple-comparisons test to identify differences within the same treatment group over time. When there was significant change over time ANCOVA was used to analyse difference between groups after adjusting for significant differences at the baseline. Statistical software SAS 9.1 (SAS Institute, Inc.) was used throughout, and *P* < 0.05 was considered to indicate statistical significance.

## 3. Results

After screening for inclusion criteria, a total of 60 subjects were recruited. Mean age was 49 years (range 40–60) and mean BMI was 28.79 kg/m^2^ (range 27–30). There were 30 females and 30 males. None of the subjects had diabetes or hypertension. Nevertheless, lipid abnormalities (triglycerides ≥ 150 mg/dL and HDL < 40 and < 50 mg/dL in men and women, resp.) and insulin resistance (<40 in males and <50 in females) were prevalent in 23.3% and 18.2%, respectively. Low HDL cholesterol (<40 in males and <50 in females) alone was prevalent in 88.3%. Mean ± SD total cholesterol, triglycerides, LDL, and HDL were 186.0 ± 42.83, 131.2 ± 66.60, 124.0 ± 42.75, and 34.6 ± 8.18 mg/dL, respectively. Mean ± SD fasting blood glucose and insulin concentrations were 87.9 ± 8.18 mg/dL and 18.2 ± 1.35 U/mL, respectively. HsCRP levels were elevated (>3 mg/L) in 83% mean ± SD 5.7 ± 2.21 mg/L. All the inflammatory markers (IL-1*β*, TNF-*α*, and IL-6) except hsCRP were maintained at low levels, but the baseline inflammatory markers including hsCRP were positively correlated (*P* < 0.05) with LDL, VLDL, triglyceride, and total cholesterol and negatively correlated with HDL. Moreover, hsCRP and IL-1*β* and TNF-*α* and IL-6 positively correlated with insulin resistance (*P* < 0.05).

The baseline characteristics and biochemical parameters of the subjects are given in Table 1. The baseline fasting blood glucose, insulin levels, and hsCRP concentration were comparable between groups. Insulin resistance as measured by HOMA was also comparable. As for the lipid profile, the baseline HDL level was significantly lower in the probiotic group and LDL was significantly higher in the probiotic + omega-3 group. Triglycerides and VLDL were comparable.

Total fecal aerobes and anaerobes (means ± SE) were 6.0 × 10^7^ ± 0.076 and 3.4 × 10^9^ ± 0.169, respectively. Total lactobacillus, bifidobacteria, streptococcus, coliforms,* E. coli,* and bacteroides were 6.3 × 10^6^ ± 0.121, 5.8 × 10^8^ ± 0.167, 5.1 × 10^2^ ± 0.148, 4.5 × 10^6^ ± 0.196, 4.1 × 10^6^ ± 0.122, and 7.9 × 10^6^ ± 0.325, respectively. Subjects with lipid abnormalities had lower total lactobacilli, bifidobacteria, and streptococcus and higher* E. coli* and bacteroides. A similar trend was observed when subjects were categorized as those with insulin resistance and those without insulin resistance based on HOMA (3.6). Also, subjects with more than 3 mg/L hsCRP had significantly lower lactobacilli, bifidobacteria, and streptococcus and higher* E. coli* when compared with those who had less than 3 mg/L.

### 3.1. Inflammatory Markers after Probiotic Supplementation

The baseline mean ± SE of hsCRP was comparable between the groups (Table 1). Mean hsCRP reduced significantly with probiotic (*P* < 0.01), and there was 24.5% and 34.6% reduction in hsCRP with probiotics (*P* < 0.01) and probiotic plus omega-3 (*P* < 0.01) groups, respectively (Table 1). Though there was a modest decrease in hsCRP with omega-3, there was no significant change when compared to the placebo group. When we assessed the relative change versus baseline, probiotics and probiotic + omega-3 were significantly different from the placebo (Table 1). There was modest reduction in proinflammatory cytokines, IL-1*β*, TNF-*α*, and IL-6 with probiotics and probiotic + omega-3.

### 3.2. Lipid Profile after Probiotic Supplementation

In the probiotic group HDL increased by 18.5% (*P* < 0.01); LDL (*P* < 0.05), triglycerides, and VLDL (*P* < 0.01) decreased by 7.04%, 5.8%, and 12.98%, respectively (Table 1). In the omega-3 group, total cholesterol, triglycerides, LDL, and VLDL decreased and HDL increased significantly by 6.7% (*P* < 0.01). HDL increased by 23.2% and LDL decreased by 10.7%; triglycerides decreased by 7.78% and VLDL by 7.78% (*P* < 0.01) in the probiotic with omega-3 group, compared with baseline levels (Table 1). The relative change versus baseline in probiotics was significantly different from the placebo group and probiotic + omega-3 (Table 1).

### 3.3. Insulin Resistance after Probiotic Supplementation

Fasting blood glucose (FBG) rose slightly in the placebo group but reduced significantly (*P* < 0.05) in the probiotic, omega-3, and probiotic + omega-3 combination groups. Similarly, the insulin levels reduced significantly (*P* < 0.05) in the probiotic, omega-3 and probiotic + omega-3 combination groups (Table 1). 

When ≥2.5 was considered as the cut-off point for insulin resistance (IR) by HOMA, all the subjects before and after supplementation were insulin resistant. However, the mean insulin resistance, which was 4.0 ± 0.3 in the probiotic group, decreased to 3.4 ± 0.04 (*P* < 0.05) after treatment. Similarly, there was a modest improvement in insulin sensitivity in the probiotic + omega-3 combination group. When ≥3.6 was considered as the cut-off point for insulin resistance (IR), all 15 subjects in the probiotic group were resistant at baseline, which reduced to 86.% (13/15) after treatment with probiotic. In the omega-3 group 86.6% (13/15) subjects were IR which was reduced (*P* < 0.05) to 46.6% (7/15) after treatment. In the probiotic and omega-3 combination group 80% (12/15) subjects were IR (≥3.6), but none were IR (≥3.6) after treatment (*P* < 0.01). In the placebo group 73% (11/15) were IR before treatment, and 93% (14/15) were IR after treatment. When we assessed the relative change versus the baseline, the probiotic group and the probiotic + omega-3 groups were significantly different from the placebo and omega-3 group.

### 3.4. Atherogenic Index

Atherogenic index was significantly (*P* < 0.01) decreased in the probiotic, omega-3, and combination groups.

### 3.5. Stool Microbiota after Probiotics and Omega-3 Supplementation

There was a significant increase in the concentration of total aerobes, total anaerobes, lactobacillus, bifidobacteria, and streptococcus in the probiotic group and probiotic + omega-3 supplemented groups. In the probiotic + omega-3 group there was a significant effect on bacteroides, coliforms, and* E. coli* as well. In the omega-3 group there was no effect on gut microbiota (Table 2).

## 4. Discussion

This randomized, controlled clinical trial showed that the probiotic preparation VSL#3 affected insulin sensitivity, lipid profile, and atherogenic index favourably and reduced hsCRP, a marker of inflammation, in overweight/obese adults. Probiotic given in combination with omega-3 was more effective than probiotic alone. Omega 3 intake is usually low in Indian population. However, omega-3 supplementation showed only marginal effects on all the parameters. Nevertheless, when it was given along with probiotic, the beneficial effect of VSL#3 observed was enhanced.

Human studies evaluating the hypocholesterolemic potential of probiotics showed beneficial effect both with* Lactobacillus* and* Bifidobacterium* strain [23, 24]. VSL#3 used in our study contained both lactobacilli and bifidobacteria strains, and the hypocholesterolemic effect was similar to that reported elsewhere [23–27]. Similarly, a study on 48 volunteers for a period of ten weeks showed significant reduction in serum cholesterol concentration with daily consumption of 200 gms of yoghurt containing* Lactobacillus acidophilus* L1 [23]. Increase in serum HDL was also observed with prolonged consumption of 300 g/day of yoghurt supplemented with* Lactobacillus acidophilus *145 and* B. longum *913 over 21-week period [25]. Apart from reduction in LDL-cholesterol some studies observed reduction of fibrinogen and proatherogenic markers with* Lactobacillus plantarum* [26, 27]. In the current study we observed reduction in total cholesterol and hsCRP and increase in HDL. However, there are quite a few studies that have failed to register any effect on cholesterol with probiotics consumption [28–30]. Administration of* Lactobacillus rhamnosus* LC705 did not influence blood cholesterol levels in 38 men and another study with* Lactobacillus fermentum* or* Lactobacillus acidophilus* failed to demonstrate any change in serum lipid levels in volunteers [28–30]. These controversial observations may be attributed to factors such as strains of probiotics used in the study or dosage and duration of treatment. One mechanistic study from our lab showed that cholesterol reduction was possible only with probiotics containing bile salt hydrolase (BSH) gene. BSH gene-negative probiotics had no effect on lipid profile [31, 32]. Though the present study does not show the mechanism of cholesterol reduction by VSL#3, it may be speculated that VSL#3 bacteria may contain the BSH gene that may be responsible for cholesterol reduction. Other proposed mechanisms include fermentation of dietary fibre in the large intestine, which releases short-chain fatty acids (SCFA) especially acetic and propionic acids which are absorbed in the blood, pass into the liver, and enter the metabolic pathways [33, 34]. There are other studies suggesting that the probiotics might reduce serum cholesterol levels due to their ability to compete with cholesterol for intestinal absorption [15].

Plasma cholesterol and triacylglycerol concentrations have been shown to be lowered by fish oil, which is rich in omega-3, through inhibition of triacylglycerol and VLDL synthesis in the liver [35–37]. Consumption of fish oil in comparison with vegetable oils such as safflower or olive oil reduces apolipoprotein B production [33]. The cholesterol-regulating effect was more pronounced when VSL#3 was given along with omega-3 fatty acid in the current study though the mechanism of synergistic effect needs to be explored. 

Probiotic yogurt containing* Lactobacillus acidophilus* La5 and* Bifidobacterium lactis* Bb12 improved fasting blood glucose in humans with type 2 diabetes [38]. Our study showed a similar effect on fasting glucose and insulin and improved insulin sensitivity in overweight nondiabetic subjects. Effect of VSL#3 on insulin resistance was shown by Li et al. in a mice model, which demonstrated decreased hepatic insulin resistance. Inhibition of proinflammatory cytokines that would induce insulin resistance has been proposed by Nestel et al. [37]. Effect of VSL#3 on insulin resistance could have been due to reduction in hsCRP and amelioration of inflammation as suggested elsewhere [39].

Insulin resistance and increased CRP concentrations have been shown to be significantly associated with several cardiovascular risk factors, such as age, smoking, hypertension, exercise, plasma lipids, homocysteine, and body mass index (BMI) [8]. Concerning the relationship between CRP concentration and BMI level, the Third National Health and Nutrition Examination survey found that the prevalence of elevated CRP levels (i.e., CRP concentrations ≥0.22 mg/dL) is higher both in overweight (BMI 25–29.9 kg/m^2^) and obese (BMI ≥ 30 kg/m^2^) patients than in normal weight (BMI < 25 kg/m^2^) subjects [40]. In line with this report we found elevated concentration (>3 mg/L) of hsCRP in 83% percent of our overweight subjects. It has been suggested that the association between BMI and CRP might be mediated by cytokines, such as IL-6 and TNF-*α*, which are both expressed in adipose tissue [9, 10] and are referred to as main regulators of CRP production in the liver [11, 12]. Indeed, in our study, high hsCRP and IL1*β*, IL-6, and TNF-*α* were positively correlated with lipid parameters (total cholesterol, triglyceride, LDL, and VLDL) and insulin resistance and negatively correlated with HDL, similar to that observed by Yudkin et al., 1999, in a study of 107 subjects [13].

In addition, the subclinical inflammation that plays a central role in most of the chronic noncommunicable diseases, such as diabetes type 2, has been shown to be linked with the composition of intestinal gut microbiota [41, 42]. Some studies have reported improvement in the mucosal barrier function and decreased intestinal endotoxin levels with bifidobacteria, leading to reduction in systemic inflammation and subsequent reduction in the incidence of diabetes [41]. In line with this, we found lower lactobacilli, bifidobacteria, and streptococcus and higher* E. coli* and bacteroides in subjects with insulin resistance (HOMA = 3.6), higher HDL and those with higher hsCRP (3 mg/L).

The value of using a single-strain probiotic over a combination of probiotic strains or species is a topic of ongoing debate. VSL#3 preparation is a cocktail of eight different probiotics, thus leaving the question of which specific probiotic(s) might be responsible for the beneficial effects described in this study. Nevertheless, the study provides important leads to conduct large clinical trials in subjects with diabetes.

To summarize, we found improved HDL, insulin sensitivity, and amelioration of inflammation (hsCRP). The study also showed increase in lactobacilli and bifidobacteria and reduction in gram negative bacteria with VSL#3 supplementation; nevertheless, improvement in insulin sensitivity and reduction in hsCRP with probiotic + omega-3 was greater than probiotic alone.

## Figures and Tables

**Table 1:** Lipids, inflammatory markers, and insulin sensitivity after treatment.

	Placebo		Probiotic		Omega-3		Probiotic + omega-3	
	Pretreatment	Posttreatment	Pretreatment	Posttreatment	Pretreatment	Posttreatment	Pretreatment	Posttreatment
Fasting blood glucose (mg/dL)	89.30 ± 3.3	91.92 ± 3.18^∗a^	88 ± 1.01	79.38 ± 1.05^∗b^	87.60 ± 1.4	83.93 ± 1.23^∗c^	86.70 ± 2.15	74.46 ± 1.188^∗cd^
Cholesterol (mg/dL)	197.80 ± 13.92	197.91 ± 13.93^a^	165.10 ± 5.76	156.06 ± 5.85^∗a^	165.50 ± 9.73	161.06 ± 9.74^∗a^	215.10 ± 7.86	204.46 ± 7.73^∗a^
Triglyceride (mg/dL)	128 ± 2.26	128.85 ± 27.7^a^	140.70 ± 17.6	133.13 ± 17.39^∗ab^	105.90 ± 6.53	102.62 ± 6.44^∗a^	150.00 ± 8.49	138.74 ± 8.35^∗b^
LDL (mg/dL)	136.30 ± 14.19	136.67 ± 14.2^a^	102.80 ± 4.91	94.50 ± 4.31^b^	106.10 ± 10.1	99.87 ± 10.08^∗b^	150.30 ± 8.41	134.58 ± 7.93^∗a^
HDL (mg/dL)	35.90 ± 2.85	35.46 ± 2.85^a^	29.60 ± 1.12	34.93 ± 1.08^∗a^	38.20 ± 1.65	40.66 ± 1.62^∗b^	34.80 ± 2.23	42.13 ± 1.89^∗b^
VLDL (mg/dL)	25.60 ± 5.45	25.77 ± 5.54^a^	32.60 ± 4.38	26.62 ± 3.47^a^	21.10 ± 1.3	20.51 ± 1.28^∗a^	30.00 ± 1.69	27.74 ± 1.67^∗a^
Atherogenic factor	196.80 ± 13.92	196.91 ± 13.93^a^	164.10 ± 5.76	155.75 ± 5.8^a^	164.50 ± 9.73	160.06 ± 9.74^∗a^	214.10 ± 7.86	203.46 ± 7.73^∗a^
Insulin level (*μ*U/mL)	17.90 ± 0.52	18.26 ± 0.43^a^	18.40 ± 0.27	17.59 ± 0.28^∗b^	18.50 ± 0.23	17.84 ± 0.22^∗a^	18.00 ± 0.3	16.75 ± 0.25^∗b^
Insulin resistance	3.90 ± 0.1	4.11 ± 0.12^∗a^	4.00 ± 0.03	3.44 ± 0.04^∗b^	4.00 ± 0.08	3.69 ± 0.07^∗c^	3.80 ± 0.08	3.07 ± 0.04^∗d^
CRP (mg/L)	5.30 ± 0.58	5.35 ± 0.58^∗a^	5.60 ± 0.52	4.36 ± 0.49^∗a^	5.60 ± 0.74	5.26 ± 0.72^∗a^	6.20 ± 0.41	4.22 ± 0.41^∗a^

Values are mean ± SEM.

*Superscript indicates a significant difference between baseline and after treatment.

*<0.05 significantly different from the pretreatment level.

^a,b,c,d^Superscript indicates significant <0.05 difference in relative changes versus baseline compared between supplemented and placebo group.

**Table 2:** Fecal bacterial count before and after treatment.

	Placebo		Probiotic		Omega-3		Pro + Omega-3	
	Pretreatment	Posttreatment	Pretreatment	Posttreatment	Pretreatment	Posttreatment	Pretreatment	Posttreatment
Total bacteria	7.74 ± 0.21	7.71 ± 0.18^a^	8.77 ± 0.09	9.13 ± 0.18^∗b^	7.7 ± 0.19	7.78 ± 0.16^a^	8.83 ± 0.09	9.55 ± 0.12^∗∗b^
Total anaerobes	9.32 ± 0.17	9.27 ± 0.12^a^	9.66 ± 0.06	10.71 ± 0.07^∗∗b^	9.44 ± 0.18	9.54 ± 0.09^a^	9.61 ± 0.1	10.86 ± 0.05^∗∗b^
Lactobacillus	6.87 ± 0.12	6.86 ± 0.14^a^	6.77 ± 0.1	7.95 ± 0.13^∗∗b^	6.76 ± 0.26	6.8 ± 0.12^a^	6.76 ± 0.12	7.96 ± 0.12^∗∗b^
Bifidobacteria	8.89 ± 0.17	8.88 ± 0.14^a^	8.71 ± 0.11	9.94 ± 0.01^∗∗b^	8.71 ± 0.08	8.75 ± 0.17^a^	8.71 ± 0.08	9.9 ± 0.03^∗∗b^
Streptococcus	2.81 ± 0.21	2.80 ± 0.31^a^	2.66 ± 0.1	8.96 ± 0.16^∗∗b^	2.67 ± 0.19	2.89 ± 0.29^a^	2.67 ± 0.11	8.99 ± 0.13^∗∗b^
Coliform	6.82 ± 0.19	6.83 ± 0.13^a^	6.59 ± 0.1	6.35 ± 0.2^a^	6.54 ± 0.13	6.44 ± 0.12^a^	6.99 ± 0.14	6.58 ± 0.11^∗∗a^
*E.coli *	6.59 ± 0.14	6.93 ± 0.19^∗∗a^	6.58 ± 0.09	6.47 ± 0.13^b^	6.55 ± 0.18	6.53 ± 0.09^a^	6.69 ± 0.19	6.39 ± 0.09^b^
Bacteroides	6.61 ± 0.19	6.98 ± 0.15^∗∗a^	6.99 ± 0.18	7.57 ± 0.17^∗∗b^	6.59 ± 0.1	6.45 ± 0.25^c^	6.96 ± 0.15	7.87 ± 0.11^∗∗d^

*<0.05 significantly different from the pretreatment level.

**<0.01 significantly different from the pretreatment level.

^a,b,c,d^Superscript indicates significant <0.05 difference in relative changes versus baseline compared between supplemented and placebo group.

Bacterial count (log⁡10 CFU/g fecal dry weight ± standard error mean).
